# Accuracy of Fully Automated 3D Imaging System for Child Anthropometry in a Low-Resource Setting: Effectiveness Evaluation in Malakal, South Sudan

**DOI:** 10.2196/40066

**Published:** 2022-10-21

**Authors:** Eva Leidman, Muhammad Ali Jatoi, Iris Bollemeijer, Jennifer Majer, Shannon Doocy

**Affiliations:** 1 Department of International Health Johns Hopkins Bloomberg School of Public Health Baltimore, MD United States; 2 International Medical Corps Juba; 3 International Medical Corps Los Angeles, CA United States

**Keywords:** mobile health, mHealth, child nutrition, anthropometry, 3D imaging, imaging, accuracy, measurement, child stature, software, algorithm, automated, device, child health, pediatric health, height, length, arm circumference

## Abstract

**Background:**

Adoption of 3D imaging systems in humanitarian settings requires accuracy comparable with manual measurement notwithstanding additional constraints associated with austere settings.

**Objective:**

This study aimed to evaluate the accuracy of child stature and mid–upper arm circumference (MUAC) measurements produced by the AutoAnthro 3D imaging system (third generation) developed by Body Surface Translations Inc.

**Methods:**

A study of device accuracy was embedded within a 2-stage cluster survey at the Malakal Protection of Civilians site in South Sudan conducted between September 2021 and October 2021. All children aged 6 to 59 months within selected households were eligible. For each child, manual measurements were obtained by 2 anthropometrists following the protocol used in the 2006 World Health Organization Child Growth Standards study. Scans were then captured by a different enumerator using a Samsung Galaxy 8 phone loaded with a custom software, AutoAnthro, and an Intel RealSense 3D scanner. The scans were processed using a fully automated algorithm. A multivariate logistic regression model was fit to evaluate the adjusted odds of achieving a successful scan. The accuracy of the measurements was visually assessed using Bland-Altman plots and quantified using average bias, limits of agreement (LoAs), and the 95% precision interval for individual differences. Key informant interviews were conducted remotely with survey enumerators and Body Surface Translations Inc developers to understand challenges in beta testing, training, data acquisition and transmission.

**Results:**

Manual measurements were obtained for 539 eligible children, and scan-derived measurements were successfully processed for 234 (43.4%) of them. Caregivers of at least 10.4% (56/539) of the children refused consent for scan capture; additional scans were unsuccessfully transmitted to the server. Neither the demographic characteristics of the children (age and sex), stature, nor MUAC were associated with availability of scan-derived measurements; team was significantly associated (*P*<.001). The average bias of scan-derived measurements in cm was −0.5 (95% CI −2.0 to 1.0) for stature and 0.7 (95% CI 0.4-1.0) for MUAC. For stature, the 95% LoA was −23.9 cm to 22.9 cm. For MUAC, the 95% LoA was −4.0 cm to 5.4 cm. All accuracy metrics varied considerably by team. The COVID-19 pandemic–related physical distancing and travel policies limited testing to validate the device algorithm and prevented developers from conducting in-person training and field oversight, negatively affecting the quality of scan capture, processing, and transmission.

**Conclusions:**

Scan-derived measurements were not sufficiently accurate for the widespread adoption of the current technology. Although the software shows promise, further investments in the software algorithms are needed to address issues with scan transmission and extreme field contexts as well as to enable improved field supervision. Differences in accuracy by team provide evidence that investment in training may also improve performance.

## Introduction

### Background

Anthropometric measurement of children is a standard component of pediatric care to enable growth monitoring as well as population-level assessments and clinical research. Despite widespread reliance on anthropometry, there has been limited technological advancement in measurement equipment. The accuracy of weight measurement was improved with the transition from spring to digital scales in the 1980s [1,2]. However, until recently, measurements of recumbent length and standing height have not benefited from similar innovations; commonly used stadiometers, or height boards, are heavy, wooden devices that are robust to field conditions but inconvenient to transport, as is commonly done for field surveys and community screenings in low-resource settings.

In recent years, 2 different types of mobile device–based technologies have been proposed as alternatives to manual anthropometry using stadiometers: (1) apps using geometric morphometric models and (2) apps using 3D imaging systems. Using a portable camera attached to a standard tablet and preloaded software, these imaging systems are able to estimate child stature (length or height), head circumference, and mid–upper arm circumference (MUAC) from a 3D model developed from a series of image captures. Geometric morphometric models aim to directly classify a child as severely or moderately acutely malnourished; examples include the Severe Acute Malnutrition Photo Diagnosis App [3] developed by Action Against Hunger Spain and the Methods of Extremely Rapid Observation of Nutrition Status developed by Kimetrica [4]. In contrast, 3D imaging system technology—currently used by the Child Growth Monitor, developed by the nonprofit Welthungerhilfe, and AutoAnthro, developed by the company Body Surface Translations Inc (BST)—produces estimates of anthropometric measurements, which can then be used to characterize nutrition status.

Preliminary validation studies for software aiming to directly classify acute malnutrition have encountered methodological as well as logistic challenges. The Photo Diagnosis App validation phase study in Spain and Senegal found high accuracy of diagnosis but suggested significant morphometric differences among the populations sampled, implying a need to investigate this morphological variability [5,6]. The researchers involved in the study noted that, although morphological variability could likely be overcome with machine learning, the approach proved very expensive compared with current technologies and that capturing a viable scan required conditions that could not be repeated in the field (AV Brizuela, personal communication, February 25, 2022). Lower accuracy was obtained by the Methods of Extremely Rapid Observation of Nutrition Status software during an initial pilot in Kenya; work is ongoing to improve performance with further calibration using a larger, multicountry data set [4].

Although the Child Growth Monitor is still in development and beta testing, several studies have evaluated the performance of AutoAnthro. Initial efficacy studies demonstrated that, in a controlled setting in Georgia, United States, devices were able to achieve high precision—the reliability of repeated 3D scans was within 1 mm of manual measurement for stature, head circumference, and MUAC; however, systematic biases were reported [7]. Replication studies in Guatemala, Kenya, and China aimed to determine whether the systematic biases observed were generalizable across populations and, therefore, something that could be corrected analytically. However, the multicountry replication studies found lower accuracy and variability in the direction and magnitude of bias [8].

Further testing in a humanitarian setting was proposed given the additional challenges for nutrition surveillance in these locations. Settings hosting internally displaced persons and refugees are commonly remote, experience austere weather conditions, and have limited or no internet connectivity. These conditions present unique operating challenges for training and use of 3D imaging technology. Changes to the software and hardware were implemented to ensure that the device and operating software were robust to and acceptable in these conditions. In addition, the timing of the evaluation—during the acute phase of the COVID-19 pandemic—presented new challenges. Travel and movement restrictions necessitated a more autonomous operation. In addition, concern about transmission risk associated with the physical contact required for traditional anthropometry, particularly height and length measurement, created additional interest in the potential for 3D imaging technology.

### Objectives

Widespread adoption of the AutoAnthro technology in humanitarian settings requires accuracy at least comparable with manual measurement notwithstanding additional constraints. Therefore, this study aimed to re-evaluate device accuracy following modifications to the software algorithm.

## Methods

### Overview

This study evaluated the accuracy of the third generation of the AutoAnthro 3D imaging system in comparison with manual measurements for child anthropometry. The third-generation software contained major updates to the previous version that were designed to achieve higher levels of durability and portability required in austere settings, improve user experience with scan capture and device performance, automate image processing, and implement changes to allow the software to operate on lower-cost hardware. Details on the hardware, positioning, data capture, and processing for the AutoAnthro technology used in this study and previous versions are compared in Table 1. Given the interest in the field-readiness of the technology, the study was embedded within a population-representative household nutrition survey conducted by International Medical Corps (IMC) to simulate the level of automation required to enable use by nonresearch actors in nutrition surveys [9]. The survey was undertaken in late 2021 (September 27 to October 2) at the Malakal Protection of Civilians site, which hosts approximately 34,000 internally displaced people and is located in the northeast of South Sudan [10].

**Table 1:** Hardware, data acquisition, review, and processing used by AutoAnthro technology to produce automated measurements of anthropometry for children.

	First generation	Second generation	Third generation
Hardware	iPad and a structure sensor 3D scanner	iPad and a structure sensor 3D scanner	Samsung Galaxy 8 phone running Android and an Intel RealSense 3D scanner
Positioning	Enumerators were unable to constrain the child’s hands or feet to help position them	Enumerators were able to constrain the child’s hands or feet to help position them	Enumerators were able to constrain the child’s hands or feet to help position them
Real-time estimates	Not available	Not available	Available
Number of scans	Unlimited scans	Fixed number of scans automatically captured	Fixed number of scans automatically captured
Data acquisition	Automatically uploaded to a computer server	Automatically uploaded to a computer server	Automatically uploaded to a computer server
Data review	Scans manually screened for data quality by enumerators	No manual screening by enumerators	No manual screening by enumerators
Data processing	Semiautomatic	Fully automatic	Fully automatic
Evidence on performance	Initial efficacy study in the United States [7]	Replication studies in Guatemala, Kenya, and China [8]	Used in this study in South Sudan

### Study Design and Data Collection

Households were sampled using a 2-stage cluster sampling design in which camp blocks were selected with probability proportional to size. Selected blocks were fully enumerated, and households were randomly selected using systematic random sampling. A sample of 485 children was targeted to achieve desired precision for estimating prevalence of global acute malnutrition, the primary survey aim. This sample was determined to be sufficient to detect a difference of 0.17 cm for height/length and 0.09 cm for MUAC given an α of .05, power of 0.8, and SDs observed in previous studies [5]. All children aged 6 to 59 months within selected households whose primary caregiver gave verbal informed consent were eligible to participate.

Staff from BST remotely trained the IMC survey manager; training included instructions on the positioning of children, use of the hardware and AutoAnthro software, and performing and saving scans. The survey manager replicated the training in person for the enumerators. Manual anthropometrics and 3D scan teams received a 4-day training. Teams jointly participated in a classroom training on study objectives and manual anthropometry. Practical exercises and a standardization test were organized separately for manual anthropometrists and scanners. All manual measurers passed the standardization test with an intra- and interenumerator technical error of measurement (TEM) for manual measurement of <1.4 for height/length and <3.0 for MUAC.

**Figure 1:**
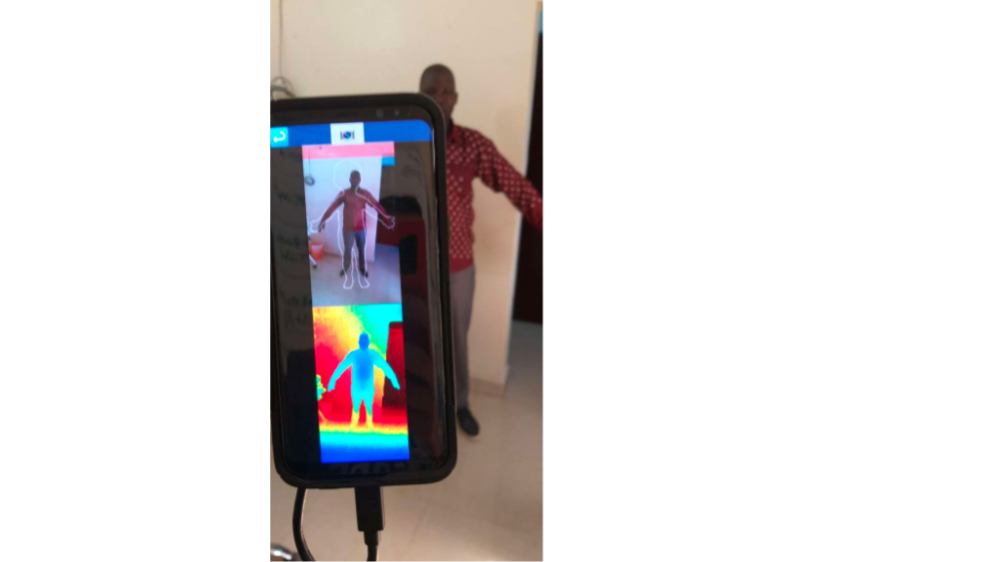
Example scan capture using the AutoAnthro software (version 3; Body Surface Translations Inc) on a Samsung Galaxy 8 Android phone and an Intel RealSense 3D scanner.

Measurements were performed by 6 teams of 4 individuals, including 2 (50%) measurers, 1 (25%) team leader trained on manual anthropometry, and 1 (25%) measurer trained to obtain the 3D scan-derived measurements. For a given child, manual measurements (weight, height/length, and MUAC) were separately obtained by 2 manual anthropometrists and entered into a survey programmed in Open Data Kit (Get ODK) on a tablet device. Anthropometrists first collected weight and MUAC; height or length was then collected following the protocol used for the 2006 World Health Organization (WHO) Child Growth Standards study [11]. After manual measurement, scans were taken by a different enumerator to ensure independence. Scans were captured using the AutoAnthro (version 3) software on a Samsung Galaxy 8 Android phone and an Intel RealSense 3D scanner (Figure 1). Each 3D imaging session comprised 10 scans, with 5 scans of both the front and back of the child. Two sets of measurements were produced: (1) a real-time, offline estimate of height/length and MUAC, which was produced by the app and displayed to data collection teams while in households for plausibility checks, and (2) an updated measurement. For the updated measurement, scan data were uploaded to a cloud server for fully automated processing, which is a slower, more computationally intensive, and generally more rigorous version of the phone-based algorithm with additional error checking for unanticipated positioning of the child. The software then compared the real-time result with the updated measurement and reported the more *consistent result*, which was identified by comparing the SD of the individual subscans (6-10 subscans produce 1 scan). In cases where the scan data were too poor to produce either real-time or updated measurements, AutoAnthro did not report results. For 11.9% (64/539) of the children, enumerators perceived the real-time estimate as implausible and took additional scans. If multiple scan sessions were performed for a given child, the median of all the individual scan sessions was used. Real-time scans were primarily used to evaluate whether they could be used for identification and referral of wasted children, whereas the analysis focused on the updated but still fully automated measurements.

Given a large number of lost scans following initial quality checks and data processing, in-depth interviews were conducted with enumerators (4 group interviews with a total of 12 enumerators) and BST staff (3 individual interviews) to document challenges. The interviews were conducted remotely in English from the United States using a semistructured interview guide and recorded to facilitate note taking.

### Analysis Methods

Differences in demographic characteristics, nutritional status of the children, and team number between children with and without scan-derived measurements were evaluated to assess characteristics associated with successful scan captures. For unadjusted comparisons, the statistical significance of differences was evaluated using the Kruskal-Wallis test for continuous variables and the Fisher exact test for categorical variables. A multivariate logistic regression was fit to evaluate the adjusted odds of achieving a successful scan.

The quality of anthropometric measurements was assessed using standard indicators—digit preference scores, proportion of outlier values, and SDs [12]. The digit preference score was calculated for height and MUAC applying the MONICA procedure, which adjusts the chi-square statistic according to the size of the sample and the df of the test. Digit preference scores values of 0 indicate a uniform distribution, and the values increase with a greater imbalance [13]. Weight-for-height or weight-for-length *z* score (WHZ) and height-for-age or length-for-age *z* score (HAZ) were calculated using the WHO growth standard [14]. Outliers were calculated using two approaches: (1) fixed exclusions of WHZ values of <−5 or >5 and HAZ values of <−6 or >6 and (2) flexible exclusions of WHZ and HAZ values of <−3 or >3 from the observed median. Measurements outside the range for which *z* scores could be generated (length: 45-110 cm for children aged <2 years; height: 65-120 cm for children aged >2 years) were also excluded. The SDs of the MUAC, WHZ, and HAZ distributions were evaluated after exclusion of outliers.

The accuracy of the measurements was visually assessed using Bland-Altman plots [15] to evaluate whether accuracy remained constant across different child body sizes and look at random bias. For the y-axis of the Bland-Altman plots, the manual measurement was subtracted from the automated scan estimate and, for the x-axis, the mean of the manual and scan estimates was used. The average bias was assessed as a metric of systematic bias (Equation 1). The limits of agreement (LoAs), that is, the 95% precision interval for individual differences, were calculated as a metric of random bias. The Pitman test of difference in variance [16] was used to test the correlation between accuracy and size of the child. TEM, an accuracy index used to express the error margin, was calculated as described by Ulijaszek and Kerr [17] (Equation 2). Analyses were performed for all children who had both scan-derived and manual measurements as well as disaggregated based on team, sex, and age groups corresponding to measurement positioning (length: ages of 6-23 months; height: ages of 24-59 months).



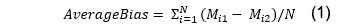





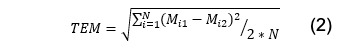



where *N* is the number of children measured, *M_i_*_1_is the scan-derived measurement for child *i*, and *M_i_*_2_is the manual measurement for the same child.

In total, 2 distinct problems with data capture were identified during the analysis. First, the AutoAnthro software estimates height, length, and MUAC by measuring the distance between the reference markers on the 3D image of the child. Visual inspection of the 3D images used to generate scan values suggested that, in select instances, the software identified a caregiver in the background, resulting in a misplaced reference marker, which typically resulted in outlier estimates. Second, the scans and manual measurements were linked using a unique ID number. Age, sex, and weight were determined for each child and entered independently by the scan-derived and manual measurement. For select children, child IDs matched across the 2 data sets, but age, sex, or weight were discordant, suggesting a potential mismatch between scan-derived and manual measurements. To evaluate the implications of these two data capture errors, Bland-Altman plots and all accuracy metrics were also calculated after excluding records with outliers or mismatches in sex, age (>6 months), or weight (>5 kg).

To evaluate the implications measurement differences would have on the classification for each derived nutrition indicator (WHZ, HAZ, and MUAC), children were classified as severely, moderately acutely malnourished, or neither using both manual and scan-derived measurements. The concordance of the classification was tabulated and visually explored. For WHZ and HAZ, values of <−3 were considered severe, and values between ≥−3 and <−2 were considered moderate. For MUAC, values of <11.5 cm were considered severe, and values of ≥11.5 cm to <12.5 cm were considered moderate. All quantitative analyses were performed in RStudio (version 1.1.456 20; R Foundation for Statistical Computing). For the qualitative analysis, detailed notes were taken during in-depth interviews, supported by automated transcription available from the Microsoft Teams software, and reviewed to synthesize key themes. The results were triangulated with the quantitative data and used to interpret and explain the quantitative findings.

### Ethics Approval

Johns Hopkins Institutional Review Board approved the study as “non-human subjects research” per DHHS regulations 45 CFR 46.102. The caregivers of the children enrolled in the study and key informants provided verbal informed consent. The study data retained for analysis were deidentified. Children identified as malnourished were referred for care, and no further compensation was provided.

## Results

### Study Sample

**Figure 2:**
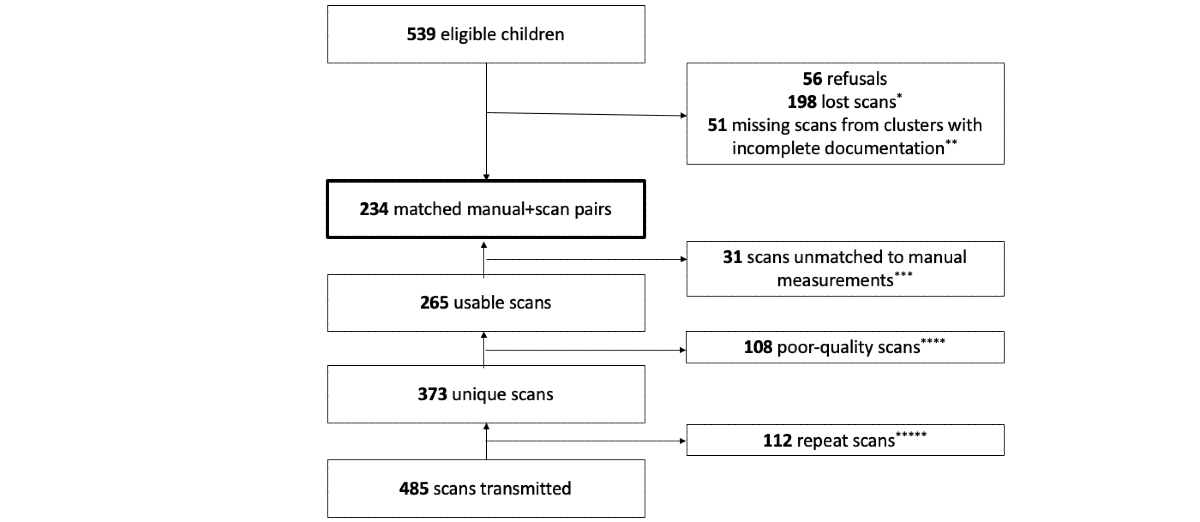
Enrollment flowchart. *Scans captured in the field that were unsuccessfully transmitted to the server and could not be recovered from devices or child identification numbers that were misreported such that scan-derived values could not be matched to manual measurements. **In five of the 32 clusters, information on the outcomes of each household visit was recorded on paper forms lost in a large rainstorm during data collection. ***The child identification number associated with the scan was not a match to any children with manual measurements. ****Scan positioning or resolution was too poor to enable calculation of scan-derived measurements. *****Field teams conducted two or more scan sessions for 64 children. When multiple scan sessions were performed for a given child, scans from all available sessions were combined, and the median of all individual scan sessions was used for the analysis.

A total of 416 households were visited, of which 325 (78.1%) had age-eligible children and consented to participate. Manual anthropometric measurements were obtained for all children aged 6 to 59 months (N=539) in the enrolled households, and scan-derived measurements were successfully processed for 43.4% (234/539) of the children. Caregivers of 10.4% (56/539) of the children refused consent for scan capture; in addition, a large number of reportedly captured scans were unsuccessfully transmitted to the server and could not be recovered from the devices (Figure 2). A total of 485 scans were successfully transmitted to the server for processing, of which 373 (76.9%) were from unique children (when multiple scan sessions were conducted, they were combined to produce a single estimate for the child). After merging sessions and removing poor-quality scans, scan-derived estimates were available for 265 individuals, of which 234 (88.3%) could be matched to manual measurements. A detailed breakdown of the available data by cluster is provided in Table S1 in Multimedia Appendix 1.

Among the final sample with both manual and scan-derived measurements, approximately equal proportions were from male participants (119/234, 50.9%) and female participants (114/234, 48.7%), and two-thirds (154/234, 65.8%) were from participants aged 24 to 59 months. The prevalence of wasting as classified by WHZ (46/234, 19.7%) exceeded that of underweight (40/234, 17.1%) or stunting (32/234, 13.7%) when using manual measurements; no children with edema were identified. When comparing the demographic characteristics and nutritional status of children with and without scan-derived measurements, there were no significant differences apart from mean age; children with both measurements were older (31.7, SD 14.9 months vs 28.9, SD 14.5 months; *P*=.03; Table 2). However, the availability of scan-derived measurements was significantly associated with team (*P*<.001), where 81% (69/85) of the children measured by team 1 had successfully transmitted scans compared with only 4% (3/81) of the children measured by team 6. Differences in the availability of scan-derived measurements by team remained significant in the multivariate logistic regression, whereas child characteristics (age, sex, height/length, and MUAC) were not associated with scan availability (Table S2 in Multimedia Appendix 1).

**Table 2:** Characteristics of the sample by availability of automated scan data (N=539).

			Children with manual measurements only (n=305)		Children with manual and scan-derived measurements (n=234)		*P* value^a^	
**Team, n (%)**								<.001
	Team 1 (n=85)	16 (18.8)		69 (81.2)				
	Team 2 (n=112)	53 (47.3)		59 (52.7)				
	Team 3 (n=106)	61 (57.5)		45 (42.5)				
	Team 4 (n=91)	78 (85.7)		13 (14.3)				
	Team 5 (n=64)	19 (29.7)		45 (70.3)				
	Team 6 (n=81)	78 (96.3)		3 (3.7)				
Age (months), mean (SD)			28.9 (14.5)		31.7 (14.9)		.03	
**Age category (months), n (%)**								.14
	6 to 23	123 (40.3)		80 (34.2)				
	24 to 59	182 (59.7)		154 (65.8)				
**Sex, n (%)**								.89
	Female	148 (48.5)		115 (49.1)				
	Male	157 (51.5)		119 (50.9)				
Underweight^b^, mean (SD)			−1.2 (1.0)		−1.2 (1.0)		.36	
**Underweight category^c^, n (%)**								.99
	Severe	13 (4.3)		10 (4.3)				
	Moderate	42 (13.8)		30 (12.8)				
Stunting^b^, mean (SD)			−0.9 (1.4)		−0.9 (1.2)		.39	
**Stunting category^c^, n (%)**								.92
	Severe	17 (5.6)		11 (4.7)				
	Moderate	32 (10.5)		21 (9)				
Wasting^b^, mean (SD)			−1.1 (1.1)		−1.0 (1.2)		.86	
**Wasting category^c^, n (%)**								.58
	Severe	10 (3.3)		9 (3.8)				
	Moderate	42 (13.8)		37 (15.8)				
MUAC^d^, mean (SD)			14.0 (1.2)		14.0 (1.3)		.52	
**MUAC category^c^, n (%)**								.52
	Severe	4 (1.3)		1 (0.4)				
	Moderate	23 (7.5)		21 (9)				

^a^Kruskal-Wallis test for continuous variables; Fisher exact test for categorical variables.

^b^Underweight was classified as weight-for-age *z* score, stunting was classified as height- or length-for-age *z* score, and wasting was classified as weight-for-height or weight-for-length *z* score using the World Health Organization growth reference based on manual measurements.

^c^For underweight, stunting, and wasting, moderate categories included *z* score values between −2 and ≥−3. The severe categories included values of <−3. For mid–upper arm circumference, the moderate category included values between 11.5 cm and 12.5 cm; values of <11.5 cm were classified as severe.

^d^MUAC: mid–upper arm circumference.

### Measurement Quality

The quality of manual measurements was evaluated for the overall sample as well as for the subset matched to scan-derived measurements. Among children with both manual and scan-derived data (234/539, 43.4%), the digit preference score for height/length was nearly twice as high for manual measurements (13.5 vs 6.8) and nearly 3 times as high (19.6 vs 6.6) for MUAC measurements as compared with scan-derived measurements, suggesting more rounding of terminal digits by manual anthropometrists. For all other quality metrics, manual measurements outperformed scan-derived measurements. For children with both measurements, no outliers were identified with fixed exclusions, and only 11 were identified with flexible exclusions (n=3, 27% for WHZ and n=8, 73% for HAZ) from the manual measurements. By comparison, for scan-derived measurements, 29 outliers were identified applying fixed exclusions (n=13, 45% for WHZ and n=16, 55% for HAZ), and 61 were identified with flexible exclusions (n=22, 36% for WHZ and n=39, 64% for HAZ). SDs were notably wider for scan-derived measurements than for manual measurements for MUAC (2.33 vs 1.26), WHZ (1.56 vs 1.16), and HAZ (1.75 vs 1.23) for all children, and the same pattern was observed for length among younger children, for whom measurement can be a greater challenge. For all quality indicators, the results were similar when quality metrics for scan-derived measurements were compared with the sample of all children (N=539) with manual measurements (Table 3).

**Table 3:** Quality of manual and scan-derived measurements as evaluated using digit preference score, outliers, and SD (N=539).

			Manual measurement				Scan-derived measurements	
			All children		All children with scans (n=234)		All children with scans (n=234)	
**Digit preference score**								
	Height or length	12.62		13.47			6.78	
	MUAC^a^	20.87		19.63		6.63		
**Outlier values (fixed)^b^, N**								
	WHZ^c^	0		0		13		
	HAZ^d^	1		0		16		
**Outlier values (flexible)^e^, N**								
	WHZ	6		3		22		
	HAZ	17		8		39		
**SD^f^ (children aged 6-59 months)**								
	MUAC	1.21		1.26		2.33		
	WHZ	1.11		1.15		1.56		
	HAZ	1.25		1.23		1.75		
**SD^f^ (children aged 6-23 months)**								
	MUAC	0.97		0.90		2.09		
	WHZ	1.19		1.28		1.59		
	HAZ	1.31		1.31		1.96		

^a^MUAC: mid–upper arm circumference.

^b^*Z* score values <−5 or >5 for weight-for-height or weight-for-length and <−6 or >6 for height- or length-for-age were considered outliers, as were measurements outside the range for which *z* scores could be generated (length: 45-110 cm for children aged <2 years; height: 65-120 cm for children aged >2 years).

^c^WHZ: weight-for-height or weight-for-length *z* score.

^d^HAZ: height-for-age or length-for-age *z* score.

^e^*Z* score values <−3 or >3 from the median *z* score of the sample for WHZ and HAZ were considered outliers, as were measurements outside the range for which *z* scores could be generated (length: 45-110 cm for children aged <2 years; height: 65-120 cm for children aged >2 years).

^f^SD calculated after excluding outlier values (weight-for-height or weight-for-length and height-for-age or length-for-age).

### Accuracy of Measurement

Analysis of scan-derived measurement accuracy used updated measurements generated after measurements were uploaded to cloud-based servers for automated processing. Real-time scan-derived measurements that were available in the field were reviewed to confirm adherence to the protocol to ensure that scan-derived measurements were not shared with manual anthropometrists (Figure S1 in Multimedia Appendix 1). Only 4 height/length and 3 MUAC manual and real-time scan-derived measurements were exact matches, providing evidence of the independence of the measurements. The correlations between manual and real-time scans were 0.54 for height/length and 0.17 for MUAC.

**Figure 3:**
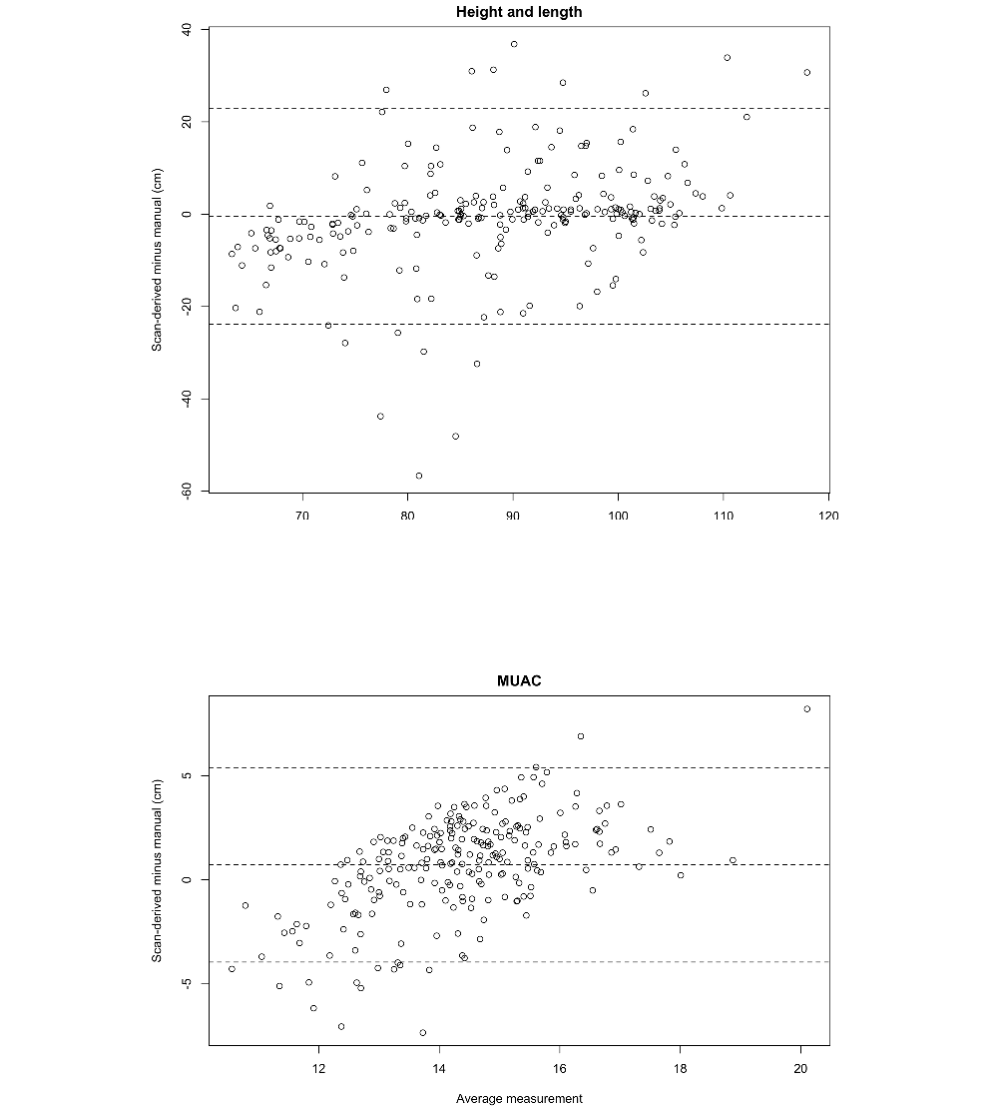
Bland-Altman plot of child stature (height and length) and mid–upper arm circumference (MUAC) comparing manual and scan-derived measurements.

Accuracy was visually inspected using Bland-Altman plots (Figure 3). When using all scans, the average bias of the measurements in cm was −0.5 (95% CI −2.0 to 1.0) for height/length and 0.7 (95% CI 0.4-1.0) for MUAC (Table 4). For height and length, 48.7% (114/234) of scan-derived measurements were higher than manual measurements or positive, and the 95% LoA was within −23.9 cm and 22.9 cm. For MUAC, 67.9% (159/234) of scan-derived measurements were higher than manual measurements, and the 95% LoA was −4.0 cm to 5.4 cm. For both indicators, the Pitman test was statistically significant (*P*<.001), suggesting differential accuracy by child size. Mean differences in height/length were negative for all children but greater for children aged 24 to 59 months compared with children aged 6 to 23 months, whereas the reverse was true for MUAC. Of interest, for height/length, the LoAs were narrower for female participants than for male participants (Figure S2 in Multimedia Appendix 1), but the mean difference was greater (−1.1 vs 0.1); the sex difference in the accuracy of MUAC was less pronounced.

Accuracy metrics varied considerably by team, excluding team 6 given the small sample of scans (n=3). For height/length, the mean difference was the greatest for team 5 (−3.8) and smallest for teams 1 (−0.2) and 3 (0.2). The width of the 95% LoAs for team 5 (−42.8 to 35.2) was nearly 3 times that of team 1. For MUAC, the mean differences were positive for teams 1 to 4 but negative for team 5, and the 95% LoAs for team 5 (−7.2 to 4.8) exceeded those of all other teams (Table 4). Differences in correlation between the manual and scan-derived measurements are visualized by team in Figure S1 in Multimedia Appendix 1.

Given the relatively wide LoAs observed in the sample overall, a sensitivity analysis was used to explore how potential errors in data capture and matching of scan-derived and manual measurements contributed to the overall accuracy (Table 4 and Figure S3 in Multimedia Appendix 1). When outlier values (n=19) and discordant pairs (n=63) were excluded, the 95% LoA was reduced (−11.9 cm to 11.4 cm for height/length and −3.5 cm to 5.1 cm for MUAC). The mean difference was reduced to −0.2 (95% CI −1.2 to 0.7) for height/length and increased marginally for MUAC; the Pitman test remained significant for both indicators.

The TEM for height/length among children of all ages was 8.4 cm; TEM is analogous to the SD, indicating that the scan-derived measurements were within –8.4 cm to +8.4 cm of manual measurements for 2 out of 3 children and within –16.8 cm to +16.8 cm for 95% of the children. The TEM for height/length was higher for children aged 24 to 59 months, male participants, and team 5. The TEM was lowest (4.2) when flagged and discordant pairs were removed. The TEM for MUAC among all children was 1.8 cm, with more limited variation by age and sex. TEM was highest for team 5 for MUAC; excluding flagged and discordant values reduced the TEM for MUAC to 1.6 cm.

**Figure 4:**
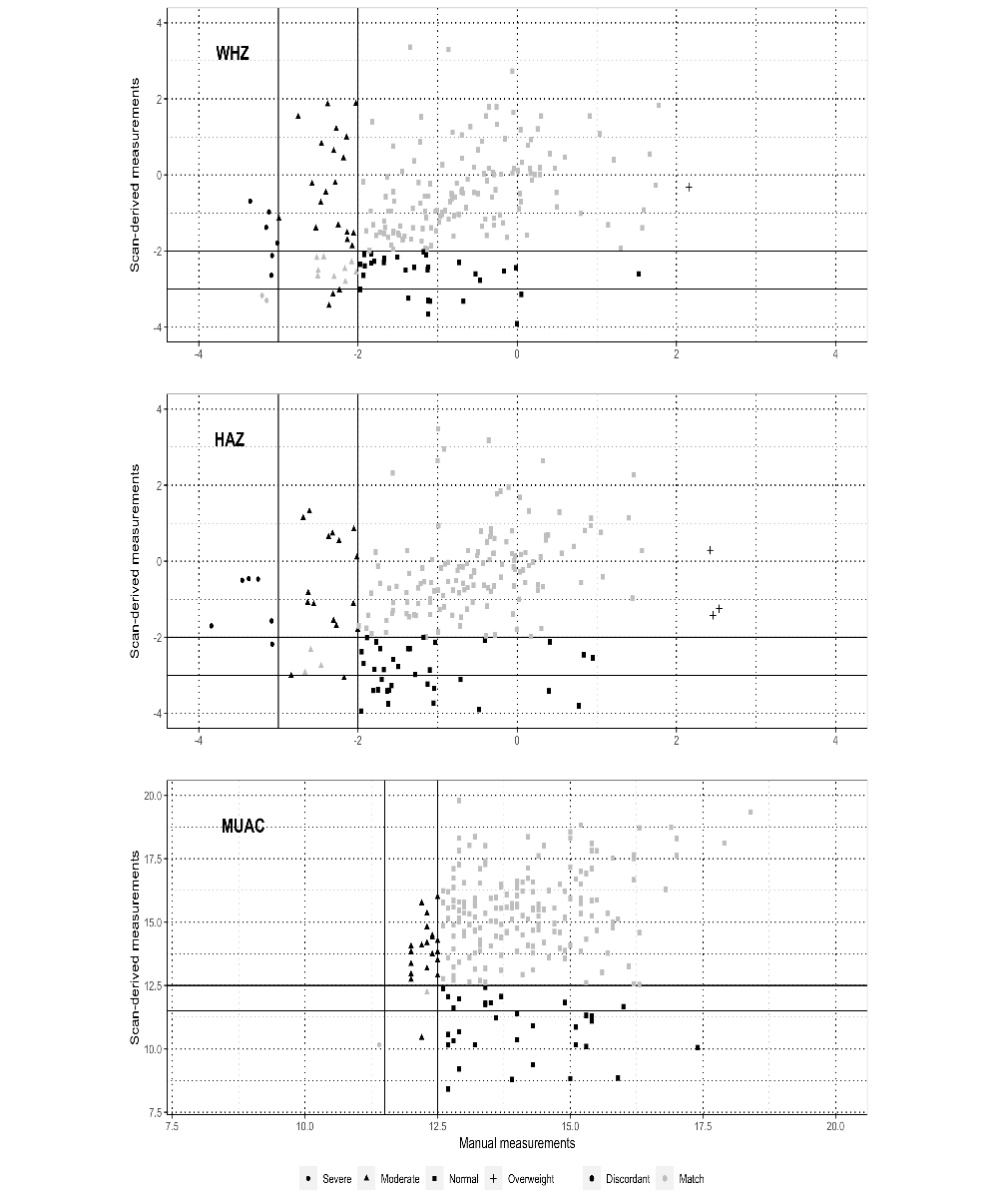
Classification of nutritional status based on manual and scan-derived measurements. HAZ: height- or length-for-age z score; MUAC: mid–upper arm circumference; WHZ: weight-for-height or weight-for-length z score.

The implications of measurement differences on classification of nutrition status were characterized for each indicator (WHZ, HAZ, and MUAC). Children were classified as severe, moderate, or normal using scan-derived and manual measurements independently, and the classifications were compared (Figure 4). Classifications were concordant for 66.7% (156/234) of the children by WHZ, 61.9% (145/234) of the children by HAZ, and 77.4% (181/234) of the children by MUAC. However, among children with low WHZ (<−2) by manual measurement, only 32% (18/56) were identified as wasted by scan-derived measurements. Similarly, among stunted (HAZ <−2) and wasted children by MUAC (MUAC <125 cm), only 23% (9/40) and 14% (3/22) were identified as stunted and wasted by scan-derived measurements, respectively.

Key informants identified several issues unique to field data collection that may have affected device performance. The following section highlights issues related to (1) beta testing or validating the device algorithm, (2) training and field supervision, (3) data capture or field work, and (4) data transmission.

**Table 4:** Statistical evaluation of differences between manual and scan-derived measurements (N=234).

				Technical error of measurement		Mean difference in cm (95% CI)		95% limits of agreement (cm)		Pitman test				Children, n (%)
										*r*		*P* value		
**Height or length**														
	All children		8.41		−0.50 (−2.03 to 1.04)		−23.86 to 22.86		0.34		<.001		234 (100)	
	Excluding flagged and discordant values^a^		4.18		−0.24 (−1.19 to 0.71)		−11.86 to 11.38		0.41		<.001		151 (64.5)	
	**Age (months)**													
		0 to 23	8.02		−0.35 (−2.88 to 2.19)		−22.7 to 22.01		0.65		<.001		80 (34.2)	
		24 to 59	8.62		−0.58 (−2.52 to 1.37)		−24.51 to 23.36		0.46		<.001		154 (65.8)	
	**Sex**													
		Female	6.85		−1.11 (−2.89 to 0.68)		−20.06 to 17.85		0.37		<.001		115 (49.1)	
		Male	9.69		0.09 (−2.41 to 2.59)		−26.88 to 27.07		0.33		<.001		119 (50.9)	
	**Team^b^**													
		Team 1	5.10		−0.15 (−1.89 to 1.60)		−14.37 to 14.08		0.20		.10		69 (29.5)	
		Team 2	7.20		0.87 (−1.79 to 3.54)		−19.19 to 20.94		0.33		.01		59 (25.2)	
		Team 3	5.64		0.15 (−2.27 to 2.57)		−15.65 to 15.94		0.33		.03		45 (19.2)	
		Team 4	8.34		−2.42 (−9.68 to 4.84)		−25.98 to 21.14		0.44		.13		13 (5.6)	
		Team 5	14.18		−3.80 (−0.978 to 2.19)		−42.83 to 35.24		0.53		<.001		45 (19.2)	
**Mid–upper arm circumference**														
	All children		1.76		0.72 (0.41 to 1.03)		−3.95 to 5.39		0.56		<.001		234 (100)	
	Excluding flagged and discordant values^a^		1.64		0.78 (0.43 to 1.14)		−3.53 to 5.10		0.51		<.001		151 (64.5)	
	**Age (months)**													
		0 to 23	1.64		0.97 (0.5 to 1.44)		−3.18 to 5.12		0.70		<.001		80 (34.2)	
		24 to 59	1.82		0.59 (0.19 to 0.99)		−4.33 to 5.50		0.58		<.001		154 (65.8)	
	**Sex**													
		Female	1.73		0.69 (0.26 to 1.13)		−3.93 to 5.32		0.54		<.001		115 (49.1)	
		Male	1.78		0.75 (0.31 to 1.19)		−3.99 to 5.48		0.57		<.001		119 (50.9)	
	**Team^b^**													
		Team 1	1.54		1.25 (0.82 to 1.68)		−2.28 to 4.78		0.55		<.001		69 (29.5)	
		Team 2	1.77		1.45 (0.91 to 1.98)		−2.60 to 5.49		0.52		<.001		59 (25.2)	
		Team 3	1.40		1.08 (0.58 to 1.59)		−2.19 to 4.35		0.41		.01		45 (19.2)	
		Team 4	1.37		0.07 (−1.14 to 1.29)		−3.86 to 4.01		0.28		.36		13 (5.6)	
		Team 5	2.30		−1.22 (−2.13 to –0.3)		−7.20 to 4.77		0.61		<.001		45 (19.2)	

^a^Records were excluded if the absolute height measurement derived from the scans was out of the range, if the weight-for-height or weight-for-length *z* score or height- or length-for-age *z* score calculated from the scanned value was considered an outlier (fixed exclusion), if the sex recorded by the manual anthropometry and the scan teams was different, or recorded ages differed by >6 months or recorded weight differed by >10 kg.

^b^Team 6 was excluded given the small number (n=3) of children with both scan-derived and manual measurements.

### Validation of Device Algorithm (Beta Testing)

The third-generation software included major software changes aimed at full automation as well as a transition to the Android platform. However, the COVID-19 pandemic and the resulting social distancing policies limited the ability of developers to test and refine the new algorithms as they had done following previous substantive revisions of the software:

We came up with this newer device, which was on an Android phone with an Intel scanner to try to provide real time results. We knew that we needed to get some preliminary data here in the US to test out the system and also to validate the algorithms and to try and make some revisions to the software you always have to come back and kind of and tweak the estimation algorithms. Because of the pandemic, we really got limited to [the developer’s] children...the kids that I really got a chance to test on were roughly from the ages of 10 to 16. So, none of the real younger kids. So, I'd say, that was really a big hold back for us. We really didn’t get to test the device rigorously before we had to send it to South Sudan and let them try and run a pilot trial.

The age of the children used for validating the algorithm may have been particularly relevant given the difference in how the software operated for younger children measured in a supine position (the software identified the child’s heel) compared with older children measured while standing (the software identified the floor). Beta testing in South Sudan revealed that the software’s ability to identify children in the supine position was more erratic. The algorithms were further adjusted before field work began. However, developers reported that further beta testing in the United States before deployment of the devices would have been valuable, particularly given the challenges in pushing software updates to South Sudan.

In addition to the demographics of the children included in the initial beta testing, pandemic-related travel restrictions meant that all prestudy testing to validate the algorithm was primarily performed in the United States in well-lit, indoor spaces. To address this, a pilot study in South Sudan was conducted in June 2021 and July 2021. Data collection in Malakal was delayed several months to allow developers time to update the software to address issues identified (eg, scan capture taking a prolonged period) before data collection. Donor deadlines prevented developers from taking more time to refine the algorithm before field-testing the updated software.

### Training and Field Supervision

South Sudan mandated a 2-week quarantine for international travelers during the study period, which prevented BST developers from traveling to conduct training and field supervision as had been done in previous surveys. Training of trainers was conducted via web-based video conferencing by the BST team from the United States, in contrast to all previous evaluations of the AutoAnthro technology. This was perceived as a major barrier as it limited the ability to quickly identify small errors in data capture during training as well as support with technical troubleshooting.

The limitations of remote training were particularly relevant during the exercise in which the enumerators practiced taking scans on children. Observing these measurements remotely proved to be impractical:

At one point we were on the phone with the group at IMC and they were trying to capture data on a child and we were getting really just garbage data. It wasn't any good at all. Not usable. And we couldn't figure out what the problem was. But it turned out that two or three of the enumerators were using the device at the same time on a child. Now if any of us had been there, we would have corrected that problem in five seconds. Because we were using a structured light approach to generate these models, when one camera is looking for its pattern, if another camera is also running at the same time, it's generating a pattern that interferes with the first one. It's a 5 second problem in person that we didn't figure out for a day or two.

During the training of enumerators, a single individual on each team was identified to be trained on the AutoAnthro technology. Enumerators felt that training all enumerators would have enabled them to better support each other; in particular, team leads (who were not trained on the software or positioning) felt unempowered to supervise the quality of the scans by their teams. In previous studies, all team members were trained on the technology:

It’s technical work. We need more training for all people...People are trained together, and some are very quick at capturing what [information] we were given in the training. In class, we are not equal...It’s good for people to be trained in one place together and then select [individuals to do scans] who would be the best to do the job.

[We] needed additional days for training on the device. [BST] needed to train all of us (not just the scanner) so we could help each other especially on positioning. More than 2 days for piloting and device training are needed. Maybe 4 or 5 days on the scanners so we have enough time to practice.

Key informants, including both enumerators and BST developers, felt that the training could have been improved by increasing the duration and improving the training materials and protocol. Although the duration of the training for this study was similar to that for previous evaluations of the AutoAnthro technology, key informants felt that, in retrospect, the training was too short. In addition, training was organized using a manual that was written in English with few photographs. As most enumerators did not speak English or spoke English as a second or third language, a more visual field manual was recommended. Finally, a standardization test (where 10 children were measured twice by each enumerator) was performed for both manual and scan-derived measurements, but only manual results were evaluated for accuracy and precision, which was perceived as a limitation.

### Data Capture

Data collection occurred during the summer period in South Sudan, where the temperature was consistently >100 °F (38 °C) and, on many days of data collection, the teams experienced a downpour of rain. When it was sunny, scans were commonly performed outside in direct sunlight. When it was raining, scans were typically performed indoors with doors and windows closed to prevent water from entering such that space and light were limited. Enumerators highlighted the small spaces and low light conditions as key barriers to obtaining successful scans:

[What we were taught was that] the result will not come accurate if the child is not positioned well, [but there was] not enough space to put this child in a good position. That is where the difference is coming from.

Limited testing was performed under these conditions; however, developers believed that neither should have affected scan performance:

You really only have to be 4 feet away from the child...When you take a picture, how far back do you normally stand? You know, I would say at least six feet. Probably more when you're taking a picture of your friends. And so [the appropriate distance for the scans] might just not be where it is natural to stand.

[Poorly lit households] should not affect scan quality. In fact, it should improve scan quality, in as much as you get rid of direct sunlight because then you can rely purely on the structured light. That should be good. [The AutoAnthro technology] has two ways of measuring distance: through this structured light by putting like a pattern on the kid and knowing where those [infrared] light dots are or [by geometry using differences in angles from] two cameras simultaneously on the object. In really bright light, you can only use the dual cameras, and you can't rely so much on the structured light.

An additional challenge noted by enumerators was that the phone frequently overheated, shut down, and gave invalid readings, an issue that was not observed in previous studies or during stress testing. However, stress testing in advance of the survey was limited to 2 hours, whereas field work lasted >8 hours per day. Key informants noted that the maximum operating temperature of the 3D scanner is 95 °F (35 °C) and that large swings in temperature can affect the trigonometry used in the scanner to assess distance (eg, affect calibration of distances between the camera and infrared light emission). However, they were uncertain regarding whether operating the devices 5 °F above the maximum operating temperature would be meaningful:

On the third day [of data collection] the device began to be hot. I reported those challenges to [the study supervisor]. The device is showing that it locked itself. When it failed, we know there was a problem. When the device failed it may bring you “00” or may give [a measurement of] 30 [cm for] MUAC...When it failed, you could click save and select end session. When the device gave you the “00,” I selected end session, [restarted] and then scanned the child again. To me it happened many times. In the third and fourth days [of data collection], maybe 3 times

each day

Scans used for analysis were automatically processed but manually reviewed for the purpose of understanding the source of errors. The 2 most common issues identified were scans captured with the feet of the child obscured (affecting height and length measurements) and with the enumerator too close to the child such that the algorithm mistook the enumerator’s arm for the child’s (affecting MUAC measurements). Unusual light conditions were also noted to have affected scans but were observed less frequently:

The feet were almost always obscured by the enumerators hand or arms. So, there were some guesswork there (I didn't touch the feet manually, but algorithmically there's going to be a lot of uncertainty as to where the feet are). In that case, you know, I think we could have written the instructions differently, meaning like just hold the child by the calves or ankles not the balls of the feet. For the older children who were standing, I saw so many cases where you couldn't see the feet at all. They were just too close, or they angled the phone incorrectly. In this example [scan from training shown] there's plenty of space between the outline of the feet in the bottom. And yet there were so many instances where you couldn't even see the child's ankles. Sometimes the hands could get cut off as well but that’s not a problem because [the algorithm] just looks for the elbows. It was rare that the...head was not captured. That happened a couple of times. It was pretty rare. The procedural problem that we saw far more often is that the enumerators arms were really close to the child's arms and that would throw a joint (e.g., move it from the child elbow to the enumerators elbow).

On the iOS device, aiming screen, you were clearly moving a cube through 3-dimensional space—that is what it looked like on the screen—and you're trying to put the kid in that cube. You had a very clear representation of what is in the cube and what is out of the cube. Your goal is to put the kid entirely in that cube. On the Android system it is much more like aiming a camera. It feels more 2-dimensional. We ended up adding a body outline which was useful, but kids were still, I think, cut off. Where with the iOS system your cut off was not the edge of the screen. You're cut off was like this aiming box within the screen. And because you're using an iPad you have a bigger kind of field of vision [compared to the Galaxy phones] is what it feels like when you're aiming it, using both hands.

Finally, enumerators noted that refusals by caregivers were very uncommon. Despite cultural sensitivities regarding capturing photographs of young children, particularly naked young children, they were typically able to reassure caregivers, showing them that 3D models (not pictures) were captured and, ultimately, most caregivers consented. However, children would sometimes cry and have tantrums and, based on this, the caregiver would withdraw consent. According to the enumerators, this was commonly observed when trying to capture scans for children aged <2 years whom they needed to lie flat on their back with arms extended, ideally separated from their caregiver.

### Data Transmission

The transmission of the data was performed by the study coordinator at the end of data collection; daily upload by enumerators was not feasible given connectivity. A total of 198 scans were lost during the transmission process. Developers have been unable to replicate the error observed during transmission such that the source of the error occurred has not been determined. On the basis of the metadata retained, developers believe it is more likely that the scans were not captured (eg, the children were positioned, but scan acquisition was not successfully initiated) than that scan transmission failed:

The data transmission is still, to my mind, somewhat of a mystery. I don't understand why we could essentially run Skype, so running video back and forth between them and our servers but we could never consistently get the data to automatically upload. We went through all kind of hoops and work arounds trying to get to make sure that we actually had all of the data. And to this day, I don't know why our software didn't work cleanly on data uploads. In other places it has worked. [In South Sudan] it works very inconsistently.

I do not believe that the data exists for a lot of those sessions. Because I could not replicate what we observed in South Sudan, where we have...a hundred children on the server, but there's only data for 20. And as far as I can tell, the only way that can happen is if you enter the child's information to create new session, but don't actually acquire the data for it.

## Discussion

### Principal Findings

This study evaluated the accuracy of scan-derived anthropometric measurements among children aged 6 to 59 months calculated using the third-generation AutoAnthro technology. This version of the AutoAnthro system aimed to optimize 3D imaging technology for adoption at scale in nonresearch settings, including austere contexts such as rural South Sudan. In contrast to previous pilots in controlled settings but consistent with other effectiveness evaluations, the quality of scan-derived measurements was substantially poorer than that of manual anthropometry [7,8]. Measurement derived from scans in our study were biologically implausible for more than 1 in 20 children applying the cutoffs recommended by the WHO [11]. Although the mean differences between measurements for both height/length and MUAC were within 1 cm, only 1 in 5 measurements of stature and 1 in 3 measurements of MUAC were within 1 cm of the manual measurement. The half width of the 95% LoAs observed was >5 times wider for MUAC and nearly 20 times wider for height/length than that observed in the previous efficacy study [7]. In addition, the magnitude of the error was associated with the size of the child such that larger errors were observed among taller children and those with greater arm circumference. The magnitude of the differences observed translated into poor classification of malnutrition; most children with wasting and stunting would not have been identified for referral using the current version of the AutoAnthro technology.

The context of the COVID-19 pandemic as well as the low-resource setting of South Sudan served to highlight logistic challenges not previously identified with the use of the 3D imaging technology and likely contributed to the low accuracy observed in our study. Successful scans were processed for only 4 in 10 children included in the study as a result of higher refusal rates, poorer scan quality, and a large number of scans unsuccessfully transmitted. Although high refusal rates have been reported in previous studies of 3D scan technologies, the magnitude of the problem in South Sudan is distinct [5,8]. COVID-19 pandemic–related social distancing orders limited the number of children, particularly younger children, measured in a supine position available to use for validating the device algorithm before the initiation of field pilots and data collection. In addition, global COVID-19 travel restrictions prevented BST developers from conducting in-person trainings or providing real-time feedback to IMC IT staff or enumerators. Both of these factors were seen as critical barriers to the success of the AutoAnthro evaluation from the perspective of the software developers and the users.

Although quantitative analysis documented accuracy too poor to support the widespread adoption of the AutoAnthro software at present, key informant interviews provided insights into investments that may improve scan capture and processing. With respect to the software platform, further enhancements are needed to ensure that scans can be transmitted successfully on low-bandwidth networks and that scans captured in extreme light conditions (direct sunlight or very low light) can be processed without issue. To support a transition to full automation, the ability of enumerators and field supervisors to review scans and metadata was more restricted than in previous versions of the technology evaluated in the studies by Conkle et al [7] and Bougma et al [8]. Although automation will be essential for adoption at scale, revisiting what information remains available to enumerators and field supervisors locally on the device may be key to ensuring that field teams can aid in guaranteeing that scans are well captured and successfully saved. In addition, empowering teams to indicate which scans should be retained for analysis may further serve to address the barriers in scan quality identified. The teams used real-time, scan-derived height/length estimates to inform whether they should collect additional scans. The analysis approach, determined a priori, used the median of all scan values without input from enumerators on field challenges. Adaptations to the algorithm that allow teams to indicate scans that should be discarded may serve to improve accuracy.

In addition, further improvements in training materials are needed to ensure a more optimal implementation without direct support from the BST team; this would ultimately be needed to allow for use at scale. Updates to training protocols and materials would benefit from translation to local languages and more illustrations to support non–English-speaking and low-literacy enumerators. Ensuring adequate time for practice is also essential. Consistent with previous research, we identified a need for further guidance on scan capture and positioning in field conditions experienced (eg, low light, small spaces, and direct sunlight) [18]. Where scans were captured with the child’s head, feet, and arms clearly in the frame and not obscured by the enumerator or caregiver, the quality was acceptable. Both the availability and accuracy of scans were strongly associated with the enumeration team, notably more so than any characteristic of the child measured. Although the study was not designed to isolate the contribution of software, hardware, and the user to device accuracy, large differences in accuracy by team help illustrate how data acquisition (eg, positioning of the child relative to the scanner and caregiver, control of lighting, adaptation to space constraints, and other environmental factors) can affect scan-derived measurements. To the extent that user variation can be controlled with additional improvements in training, this may present an opportunity for future performance improvements.

This study is subject to at least six limitations. First, scans for over one-third of the sampled children were not successfully transmitted to the cloud server and could not be recovered from the devices. Although successful transmission of scans was not associated with child demographic characteristics or nutritional status, the loss of data resulted in a smaller sample size and limited power for planned analysis. Second, to ensure that the manual and scan-derived measurements were correctly matched, the child’s age, sex, and weight were entered into both data sets as well as a child identification number. However, for many children with matched identification numbers, these other values were not a perfect match, prompting concerns about whether both measurements were truly from the same child. Third, manual measurements were used as the standard for evaluating the scan-derived measurements; however, there is some indication of terminal digit preference score, and the SD of WHZ and HAZ values exceeded 1.1, an indication of potential measurement error [11,19]. Fourth, given the humanitarian context, repeat manual or scan-derived measurements were not collected. As a result, we were unable to evaluate the reliability of these measurements. Fifth, information on the number of eligible children measured per household was recorded on paper forms. The forms for 5 clusters were damaged in a heavy rainstorm such that the total number of refusals in these clusters is unknown. Finally, the study sample is unique in that the population sampled was from a single internally displaced person site, and data collection occurred during the COVID-19 pandemic, both factors that may affect the generalizability of the findings to other populations and periods.

### Conclusions

This study was initiated given considerable interest in 3D imaging technology, the potential use of the lightweight hardware, strong user acceptability, and evidence supporting the potential time savings relative to manual anthropometry [20]. Previous studies in controlled settings provided evidence that repeated scans could reliably estimate height/length and MUAC, suggesting the potential of 3D imaging technology as an alternative to manual measurement [7]. This study aimed to evaluate whether these results could be replicated at scale with full automation of scan processing and minimal oversight of training and data collection. Enumerators communicated an overall interest in the device performing well given that the scan capture generally took less time than manual measurement and eased field work. However, our findings suggest that the scan-derived measurements produced by AutoAnthro were not of sufficient accuracy for widespread adoption. Developers generally concluded that they needed more time to test and improve training and software; pandemic and financial barriers prevented them from ensuring that the software worked as intended before final testing. Further investments in the software algorithms are needed to address issues with scan capture and transmission—to ensure that scans can be captured in difficult field contexts (eg, extreme light conditions and temperature conditions) and efficiently transmitted on low-bandwidth networks. In addition, software revisions aimed at empowering field enumerators and supervisors were proposed, including local retention of data to facilitate field review of scan capture completeness and quality. Finally, differences in accuracy by team provide evidence that investments in training may also be able to improve performance.

## Appendix

Multimedia Appendix 1 Additional tables and figures with data related to scan availability and accuracy.

## Abbreviations

BST: Body Surface Translations Inc
HAZ: height-for-age or length-for-age z score
IMC: International Medical Corps
LoA: limit of agreement
MUAC: mid–upper arm circumference
TEM: technical error of measurement
WHO: World Health Organization
WHZ: weight-for-height or weight-for-length z score
