# Supplementary material for: Accuracy of Fully Automated 3D Imaging System for Child Anthropometry in a Low-Resource Setting: Effectiveness Evaluation in Malakal, South Sudan
Source: JMIR Biomed Eng. 2022 Oct 21;7(2):e40066. doi: 10.2196/40066 (PMC11041446; doi:10.2196/40066)
Supplement: Multimedia Appendix 1 [file biomedeng_v7i2e40066_app1.docx]

**Multimedia Appendix 1**

Supplemental Table 1: Number of manual measurements, scans and scan refusals, by cluster

| **Cluster number** | **Team** | **Total Eligible Children** | **Number of Children Scanned** | **Number of Successful Processed Scans** | **Refusals** | **Lost Scans** |
| --- | --- | --- | --- | --- | --- | --- |
| 1 | 1 | 11 | 11 | 10 | 0 | 1 |
| 2 | 2 | 19 | 17 | 17 | 2^2^ | 0 |
| 3 | 3 | 13 | 13 | 12 | 0 | 1 |
| 4 | 4 | 19 | 12 | 6 | 4 | 9 |
| 5 | 5 | 13 | 12 | 12 | 1 | 0 |
| 6 | 6 | 11 | 5 | 0 | 5 | 6 |
| 7 | 1 | 20 | 15 | 19 | 1^2^ | 0 |
| 8 | 2 | 19 | 19 | 0 | 0 | 19 |
| 9 | 3 | 16 | 15 | 16 | 0^2^ | 0 |
| 10 | 4 | 17 | 13 | 0 | 2 | 15 |
| 11 | 5 | 14 | 13 | 9 | 0 | 5 |
| 12 | 6 | 20 | 14 | 0 | 4 | 16 |
| 13 | 1 | 23 | 21 | 22 | 1^2^ | 0 |
| 14 | 2 | 19 | 19 | 19 | 0^2^ | 0 |
| 15 | 3 | 14 | 14 | 1 | 0 | 13 |
| 16 | 4 | 14 | 7 | 7 | 7 | 0 |
| 17 | 5 | 10 | 10 | 5 | 1 | 4 |
| 18 | 6 | 17 | 8 | 0 | 3 | 14 |
| 19 | 1 | 14 | Unknown^1^ | 3 | Unknown^1^ | Unknown |
| 20 | 2 | 18 | Unknown^1^ | 5 | Unknown^1^ | Unknown |
| 21 | 3 | 20 | Unknown^1^ | 14 | Unknown^1^ | Unknown |
| 22 | 4 | 19 | Unknown^1^ | 0 | Unknown^1^ | Unknown |
| 23 | 5 | 12 | Unknown^1^ | 10 | Unknown^1^ | Unknown |
| 24 | 6 | 16 | 12 | 1 | 6 | 9 |
| 25 | 1 | 17 | 10 | 15 | 0 | 2 |
| 26 | 2 | 18 | 18 | 0 | 2 | 16 |
| 27 | 3 | 20 | 16 | 2 | 1 | 17 |
| 28 | 4 | 22 | 20 | 0 | 2 | 20 |
| 29 | 5 | 15 | 7 | 9 | 6^2^ | 0 |
| 30 | 6 | 17 | 10 | 2 | 6 | 9 |
| 31 | 2 | 19 | 16 | 18 | 1^2^ | 0 |
| 32 | 3 | 23 | 14 | 0 | 1 | 22 |

^1^ Information on the outcomes of each household visit were recorded on paper records lost during a large rainstorm during data collection.

^2^ Cluster revisited to encourage additional enrollment. Number reflects the number of refusals after the second visit.

Supplemental Table 2: Parameters associated with available scan derived anthropometric measurements

|  | Crude odds ratio | | | Adjusted odds ratio^1^ | | |
| --- | --- | --- | --- | --- | --- | --- |
|  | Point Estimate | 95%  Confidence Interval | p-value | Point Estimate | 95%  Confidence Interval | p-value |
| **Team** |  |  |  |  |  |  |
| Team 1 | (ref) |  |  | (ref) |  |  |
| Team 2 | 0.26 | 0.13, 0.49 | **<0.001** | 0.27 | 0.13, 0.51 | **<0.001** |
| Team 3 | 0.17 | 0.09, 0.33 | **<0.001** | 0.16 | 0.08, 0.32 | **<0.001** |
| Team 4 | 0.04 | 0.02, 0.08 | **<0.001** | 0.04 | 0.02, 0.08 | **<0.001** |
| Team 5 | 0.55 | 0.25, 1.18 | 0.12 | 0.54 | 0.25, 1.15 | 0.11 |
| Team 6 | 0.01 | 0.00, 0.03 | **<0.001** | 0.01 | 0.00, 0.03 | **<0.001** |
| **Age categories** |  |  |  |  |  |  |
| 6-23 months | (ref) |  |  | (ref) |  |  |
| 24-59 months | 1.30 | 0.91, 1.86 | 0.15 | 1.49 | 0.74, 3.04 | 0.27 |
| **Sex (Ref: Male)** |  |  |  |  |  |  |
| Female | (ref) |  |  | (ref) |  |  |
| Male | 0.98 | 0.69, 1.37 | 0.89 | 0.87 | 0.57, 1.30 | 0.49 |
| **Height** (Manual Measurement) | 1.02 | 1.00 1.03 | **0.03** | 1.00 | 0.97, 1.03 | 0.80 |
| **MUAC** (Manual Measurement) | 0.99 | 0.86, 1.15 | 0.94 | 0.85 | 0.69, 1.03 | 0.09 |

^1^ Adjusted odds ratios estimated from multivariate logistic regression model

Supplemental Figure 1: Correlation between manual measurements, real-time scan derived measurements, and final scan derived measurements, by team^1,2^

**Panel A: Height**


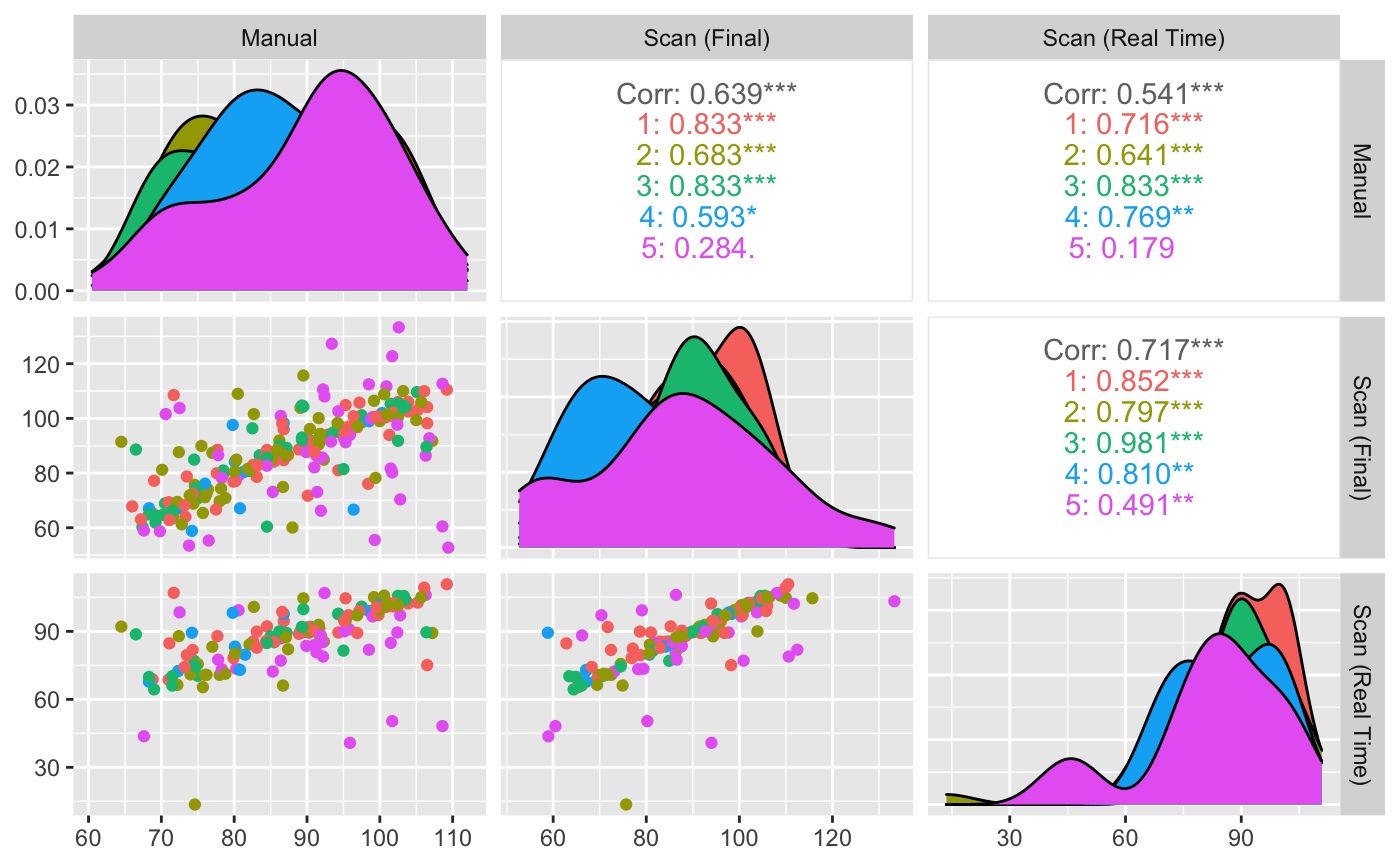


**Panel B: MUAC**


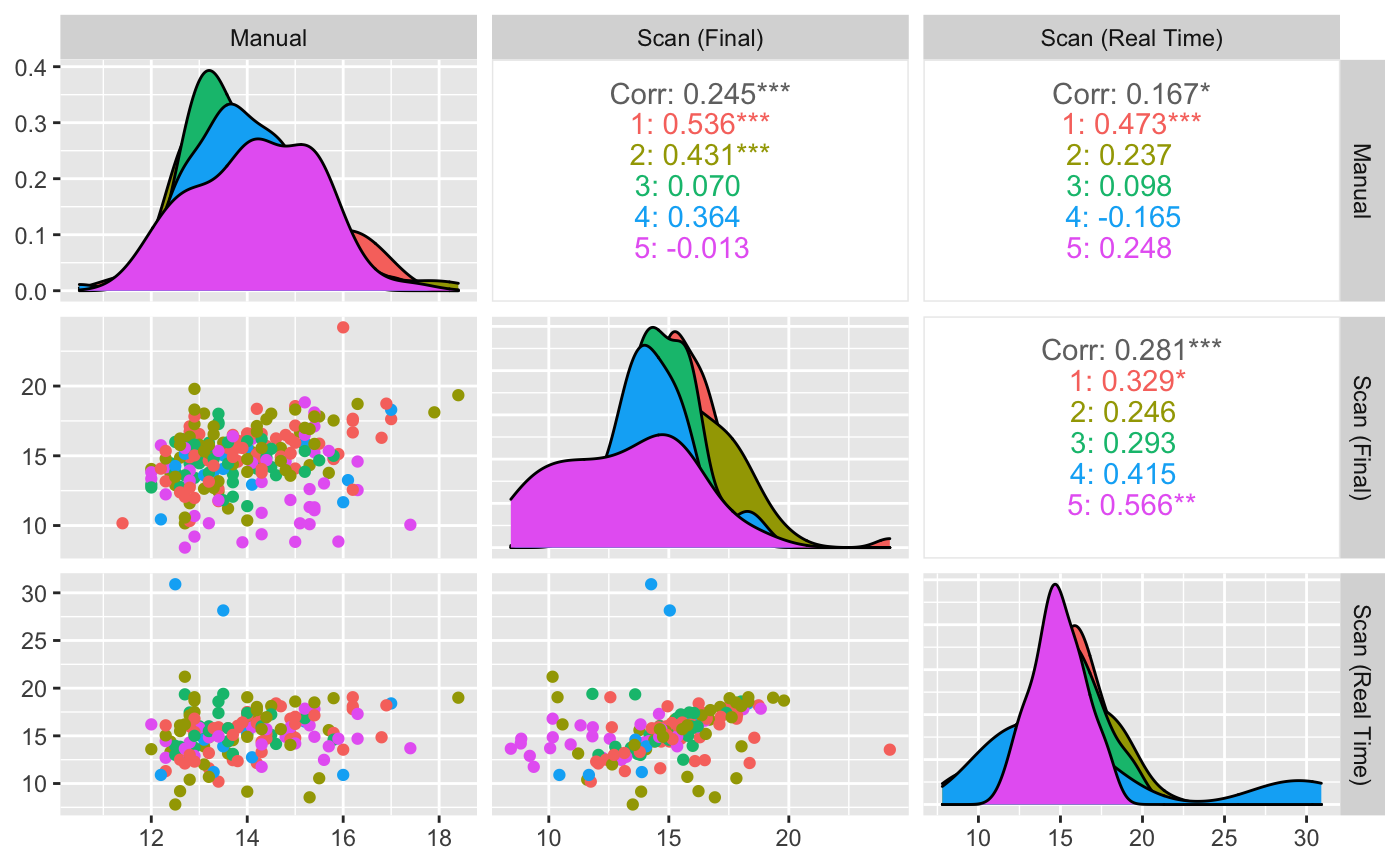


^1^ Real-time estimate were produced on the device locally at the time the scan was obtained. Final estimates used for analysis were produced from scans uploaded to a cloud server and processed using computer-based software, with additional error checking. Both methods are fully automated.

^2^ Given the small number of children with both scans and manual measurements (n=3), children measured by team 6 were excluded from figure.

Supplemental Figure 2: Bland Altman Plots by Sex

| 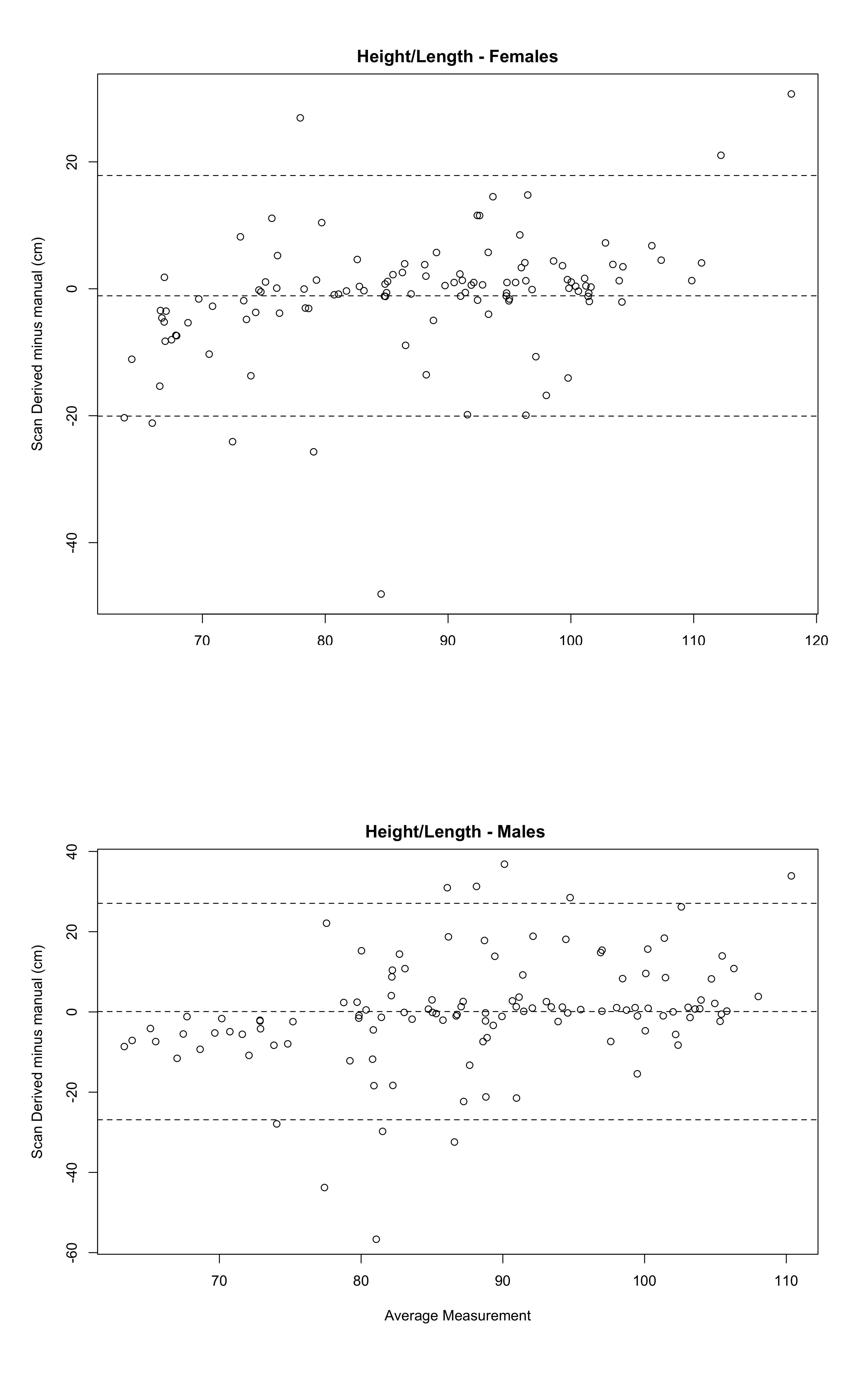 | 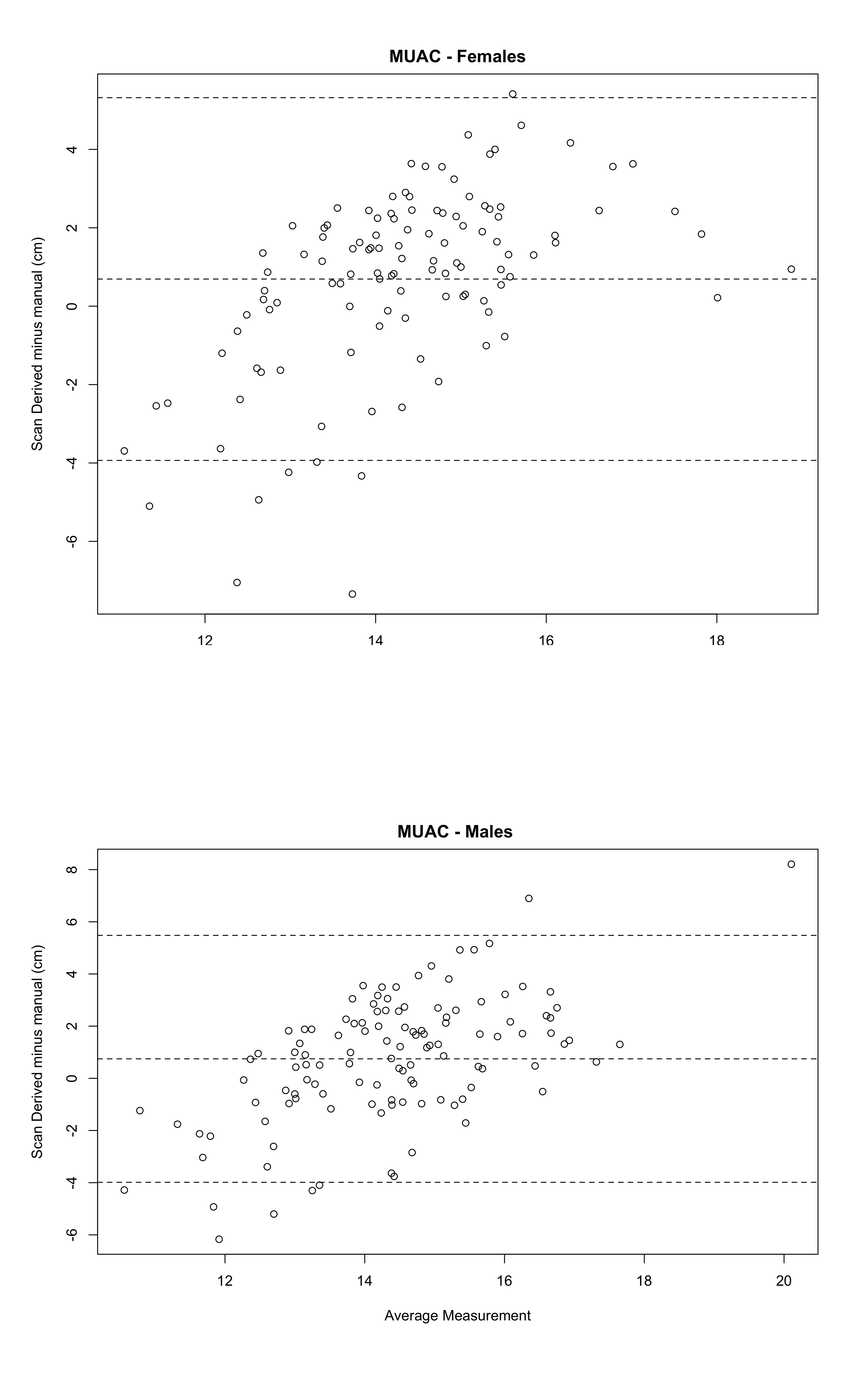 |
| --- | --- |

Supplemental Figure 3: Bland Altman Plots Excluding Flagged (n=19)^1^ and Discordant^2^ (n=63) Values


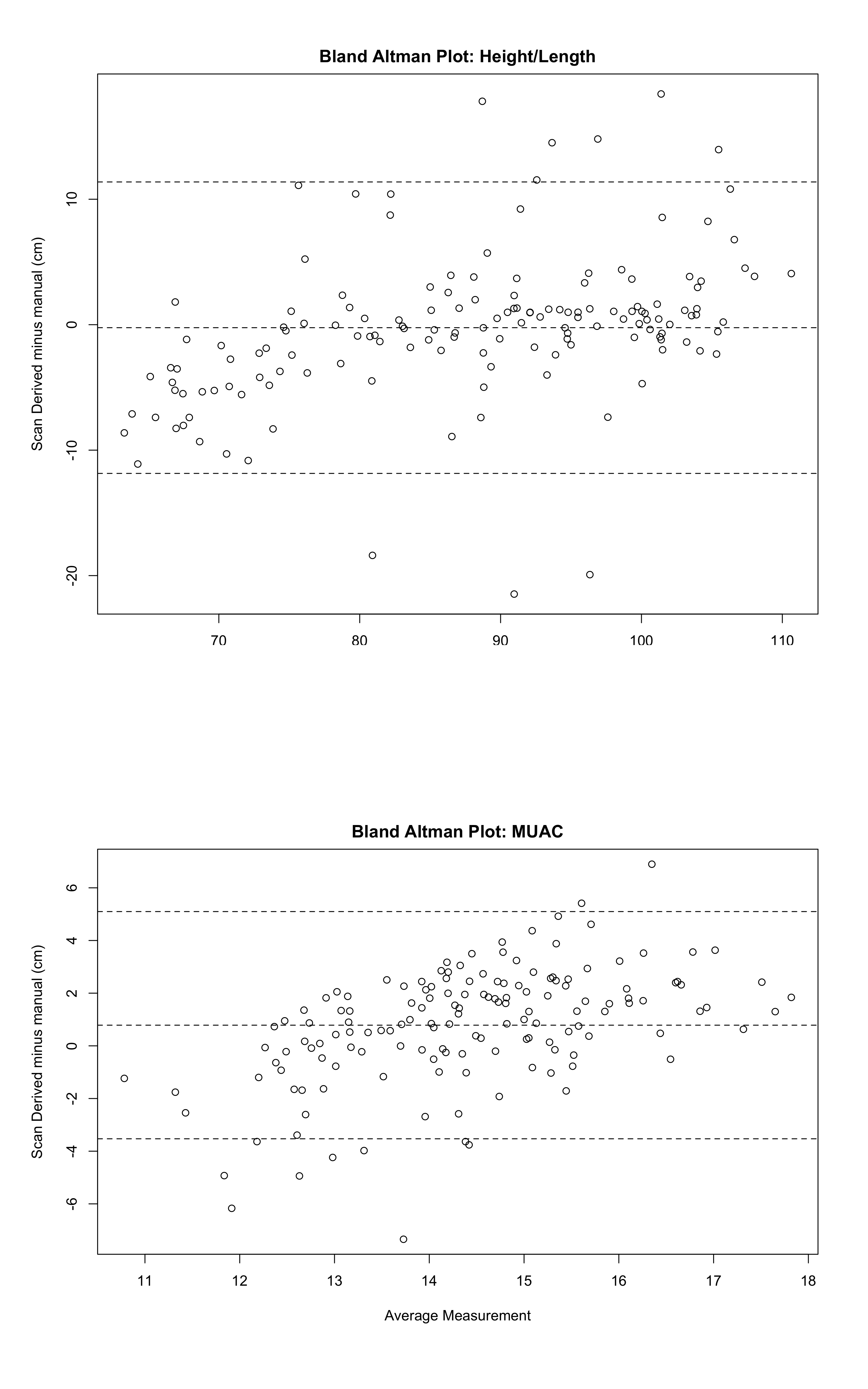


^1^ Records were excluded if the absolute height measurement derived from the scans was out of the range or if weight-for-height and/or height-for-age calculated from the scanned value was considered an outlier (fixed exclusion).

^2^ Matches were considered discordant if the sex recorded by the manual anthropometry and the scan teams were different, and/or recorded ages were more than 6 months different and/or recorded weight was more than 5kg different.
